# The attention network characteristics of adults with high ADHD traits: low stability, boost accuracy by sacrificing response time

**DOI:** 10.3389/fpsyg.2024.1477581

**Published:** 2024-12-18

**Authors:** Jie Xiang, Xueke Wang, Tingyong Feng

**Affiliations:** ^1^Faculty of Psychology, Southwest University, Chongqing, China; ^2^Key Laboratory of Cognition and Personality, Ministry of Education, Chongqing, China

**Keywords:** high ADHD traits, Adult ADHD Self-Report Scale (ASRS), attention network test (ANT), intra-individual coefficient of variation (ICV), balanced integration score (BIS)

## Abstract

Adults with high ADHD traits (H-ADHD) face challenges in academics, social interactions, and careers, yet their attention characteristics remains unclear. Using Attention Network Test (ANT), we examine attentional characteristics (including specific attentional qualities and overall attentional performance) of H-ADHD and explore how their specific attentional qualities impact overall attentional performance. We find H-ADHD primarily manifests lower alerting network, higher intra-individual coefficient of variation (ICV) and poorer balanced integration score (BIS). These results remain stable even after controlling for covariates such as anxiety and depression scores. Furthermore, the BIS deficiency in high attention deficit traits group (H-ADHD-I) specifically manifests as “high accuracy—slow reaction time,” reflecting their response pattern as the reaction time compensated accuracy. Additionally, compared to alerting network, attentional variability (ICV) has a greater role in mediating the impact of ADHD symptoms on overall attentional performance (BIS). Taken together, H-ADHD mainly exhibit deficits in sustained attention (alerting network), attentional stability (ICV), and overall attentional performance. ADHD symptoms worsen overall attentional performance due to increased attentional variability. The study emphasizes the sensitivity of alerting network, ICV, and BIS indicators, and highlights the significance of attentional variability, suggesting their potential clinical value in the future.

## 1 Introduction

Attention-Deficit/Hyperactivity Disorder (ADHD) is a neurodevelopmental disorder that presents with inattention and/or hyperactivity-impulsivity (American Psychiatric Association, 2013). It affects 8.0% of children and adolescents worldwide (Ayano et al., 2023), with about 2.58% continuing to have symptoms into adulthood (Song et al., 2021). In adults, inattention is often more prominent than hyperactivity and impulsivity. They may demonstrate slower cognitive processes, exhibit long-winded and tangential communication, and face challenges in decision-making (Kooij et al., 2019). These attention-related issues significantly impact their psychosocial functioning in various domains, including social, academic, and occupational settings, affecting overall well-being (Faraone et al., 2015; Faraone et al., 2021). Consequently, they often co-occur with emotions such as anxiety depression and stress (Riglin et al., 2021; Gair et al., 2021). Despite the common occurrence of attention deficits in adult ADHD, understanding their specific patterns remains a critical area of investigation.

Classic attention tasks, such as continuous performance tests (CPTs) and visual search, mainly focus on specific attentional aspects and may not cover all dimensions comprehensively. In contrast, Posner and colleagues proposed the Attention Network Model, categorizing attention into three networks: alerting, orienting, and executive control networks. Alerting is defined as the attainment and maintenance of an alert state, reflecting the breadth and stability of attention. Orienting involves the selection of sensory input information, indicating selective attention and the ability to shift attention. Executive control is defined as the resolution of conflicts between responses, reflecting the allocation, monitoring, and regulation of attention (Posner and Petersen, 1990; Fan et al., 2005). Building upon this model, Fan et al. (2002) developed the Attention Network Test (ANT) to measure the three networks distinctly. The ANT, along with the model, offers a more comprehensive perspective for assessing attention, addressing the limitations of other tasks (Baggio et al., 2020; McGee et al., 2000). As a result, this approach has stimulated research into attention deficits in conditions like ADHD and other neuropsychiatric disorders (Fan and Posner, 2004; Vazquez-Marrufo et al., 2019).

Existing research has indicated that adults with ADHD exhibit deficiencies in alerting (Oberlin et al., 2005) and executive control networks (Oberlin et al., 2005; Kim and Kim, 2021), increased reaction time variability (Lundervold et al., 2011; Lampe et al., 2007), slower reaction times (Oberlin et al., 2005; Lampe et al., 2007), and lower accuracy (Lundervold et al., 2011) in attention tasks. However, findings regarding the three major networks, reaction time, and accuracy in adult ADHD within the ANT are not entirely consistent. Specifically, certain studies have detected impairments in the executive control networks among adults with ADHD (Oberlin et al., 2005; Kim and Kim, 2021), whereas alternative research has failed to observe such a deficit (Lundervold et al., 2011; Coll-Martin et al., 2021). This highlights the need for further investigation into attentional network performance in adults with ADHD and the search for additional stable and sensitive indicators.

Intraindividual variability (IIV) in cognitive task performance, widely employed in ADHD research, is considered a potential internal phenotype of ADHD (Bidwell et al., 2007; Pinto et al., 2016). Its extensive application in attention research is evident (Wallace et al., 2019; Levy et al., 2018). Intra-individual coefficient of variation (ICV; calculated as SDRT/mean RT) serves as an indicator of Intra-Individual Variability (IIV) and is a common method for reflecting attentional variability which further reflects poorer attentional stability. Recognized as a critical measure of attentional control efficiency, ICV’s importance in ADHD studies is growing (Johnson et al., 2015). However, ICV is less frequently used in the context of ANT compared to classic attention tasks like CPTs.

Reaction time and accuracy are key performance indicators widely used in ADHD research. However, there’s an ongoing debate about their interpretation in ADHD attention tasks. Some studies suggest that adults with ADHD may exhibit lower accuracy but similar reaction times compared to the control group in the ANT (Lundervold et al., 2011). Conversely, other studies indicate significantly slower reaction times in adults with ADHD in the ANT compared to the control group (Lampe et al., 2007; Dotare et al., 2020), or with no significant difference in accuracy (Zamani Sani et al., 2020). Additionally, Kim and Kim (2021) found no discernible disparities in accuracy and reaction time between adults with ADHD and the control group. Moreover, longer reaction times theoretically associate with higher accuracy; relying solely on these indicators is not comprehensive, as higher accuracy may be due to longer reaction times, and the latter might be a deliberate strategy for accuracy. Some researchers have therefore proposed speed-accuracy trade-off measures (Vandierendonck, 2017; Liesefeld et al., 2015), among which the balanced integration score (BIS; calculated as *z_PC_* − *z_meanRT_*) is less affected by data distribution and performs well (Liesefeld and Janczyk, 2019; Liesefeld and Janczyk, 2023). It has already found application in visual memory search tasks and cognitive tasks involving older adults (Auerswald et al., 2022). Therefore, we incorporated the BIS index in ANT to reflect overall attentional performance and further investigated reaction time and accuracy balance in adults with ADHD.

The three attention networks and attentional variability (ICV) are fundamental attention indicators reflecting specific attentional quality, and BIS is the comprehensive indicator assessing overall attentional performance. Theoretically, poor specific attentional quality can affect overall attentional performance. Within the realm of specific attentional qualities, the alerting network, foundational among the attention networks, significantly influences overall attention, enhancing accuracy (Manly et al., 2004). Furthermore, heightened ICV is frequently associated with prolonged reaction times (Anstey, 1999; Loomans et al., 2012; Ghisletta et al., 2018) and low accuracy (Anstey, 1999). In general, poorer specific attentional quality is associated with poorer overall attentional performance (BIS). However, limited research has explored their interplay in adults with ADHD. Overall attentional performance, as the most overt manifestation, is closely related to the daily lives of adults with ADHD. Understanding mediating mechanisms between ADHD symptoms and overall attentional performance can inform targeted interventions, potentially enhancing their quality of life.

Compared to adults with ADHD, there’s been limited research on those at high traits. Understanding the ANT performance in this group is underexplored. Insights into their attentional traits can help proactively address potential issues, aiding their adaptation to daily life and work/study environments. This knowledge can also enhance diagnostic accuracy and treatment planning for clinicians, ultimately improving therapeutic outcomes. Exploring the attentional profile of high ADHD traits adults provides valuable insights into unique features compared to general adult ADHD, contributing to a deeper understanding of the disorder. Therefore, research on high ADHD traits adults is equally pivotal.

The study has two main objectives. Firstly, it examines attentional characteristics of H-ADHD by comparing high ADHD traits adults with controls in the ANT. This includes assessing fundamental attention indicators (the three networks and ICV) and a comprehensive indicator (BIS). Based on previous studies, we anticipate impairments in the alerting network, executive control network, attentional variability (ICV), and overall attentional performance (BIS) in high ADHD traits adults. Secondly, we investigate how these specific attentional qualities influence overall attentional performance. Using a mediation model, we examine their role in mediating the impact of ADHD symptoms on overall attentional performance. We expect that more severe ADHD symptoms will lead to poorer attentional qualities and, consequently, diminished overall attentional performance (BIS).

## 2 Materials and methods

### 2.1 Participants

A total of 430 participants were recruited in Chongqing, China, and they completed the Adult ADHD Self-Report Scale (ASRS) either online or offline. Among them, 9 participants did not pass the deception check questions. The remaining 421 participants were invited to participate in the Attention Network Test (ANT) task. Out of these, 374 participants completed the ANT task. However, 15 participants were excluded due to having within-subject reaction times that fell beyond 3 SDs from the mean, which reflects their seriousness across each trial. Additionally, 16 participants were excluded because their accuracy rate, which reflects their overall seriousness throughout the test, was below 80% (Frick et al., 2023). This left a sample of 343 participants (mean age = 21.00, SD = 0.91, range = 18–24 years; 101 males; Han-Chines e = 282, others = 61; Supplementary Table S1) for subsequent analysis. Within this group, when calculating attention network indices, 4 participants were excluded because their reaction times were beyond 3 SDs from the mean of all participants. When calculating overall attentional performance (BIS), 8 participants were excluded because their accuracy rates or reaction times were beyond 3 standard deviations from the mean of all participants. When calculating intra-individual coefficient of variation (ICV), 18 participants were excluded because at least one of their ICV values (including congruent/incongruent/neutral/total ICV) was beyond 3 SDs from the mean of all participants. For the mediation analysis, as participants had to meet all the selection criteria, 26 participants were excluded.

Therefore, the final sample for attention network analysis included 339 participants, the sample for speed-accuracy balance analysis included 335 participants, the sample for reaction time variability analysis included 325 participants, and the sample for mediation analysis included 317 participants. We had obtained appropriate ethics committee approval for the research reported, and all participants gave written informed consent in our experiment.

### 2.2 Attention network test

The ANT, developed by Fan et al. (2002), combines cued reaction time with the flanker task to evaluate alerting, orienting, and executive control networks, based on response times to different cues. The precise design involves a 10 min task, with each trial lasting 4,000 msec. Supplementary Figure S2A shows cue conditions, and Supplementary Figure S2B displays target stimuli. Supplementary Figure S2C illustrates a trial’s time course, including a fixed duration, warning cue, simultaneous presentation of target and flankers, response, and post-target fixation.

### 2.3 Measuring attention

The three major metrics [attention network efficiency (ANE), balanced integration score (BIS), and intra-individual coefficient of variation (ICV)] are utilized to measure attention. ANE assesses alerting, orienting, and executive control network efficiencies. Alerting network efficiency is the difference between no-cue and double-cue response times. Orienting network efficiency compares center and spatial cue conditions. Executive control network efficiency measures incongruent and congruent flanker response time differences. Additionally, alerting network 2, computed as the no-cue and center-cue response time difference, accounts for distracting stimuli. ICV is the standard deviation of reaction time divided by the mean reaction time. BIS is calculated as zPC—zmeanRT, with zPC indicating standardized accuracy rates and zmeanRT representing standardized average reaction times in the ANT task.

### 2.4 Measuring ADHD symptoms

The Adult ADHD Self-Report Scale (ASRS) is a rating scale designed to assess current ADHD symptoms, encompassing the 18 DSM-IV criteria for ADHD. Numerous studies have indicated that the ASRS demonstrates robust reliability and validity, exhibiting high consistency with clinical physician diagnoses. It is considered a reliable and effective scale for assessing adult attention-deficit/hyperactivity disorder (ADHD) symptoms and can be utilized in the clinical evaluation of adult ADHD (Alemany et al., 2015; de Zeeuw et al., 2008). Therefore, it has been introduced, translated, and applied in many countries for the screening of adult attention-deficit/hyperactivity disorder (ADHD) symptoms (Seli et al., 2012; Samyn et al., 2017; Yoon, 2010). In this study, participants rate symptoms on a 5-point scale, ranging from 0 (never/seldom) to 4 (very often), resulting in a total score range of 0 to 72. In the current study, we incorporate the total ASRS score as a continuous variable for correlation analysis. Additionally, we categorize participants into groups for inter-group comparisons based on a threshold of 17 points for each sub-dimension (Yoon, 2010; Yeh et al., 2008). These groups include: a high attention deficit traits group (H-ADHD-I; inattention dimension score ≥ 17 points) and its control group (L-I; inattention score < 17 points); a high hyperactivity-impulsivity traits group (H-ADHD-H; hyperactivity-impulsivity dimension score ≥ 17 points) and its control group (L-H; hyperactivity-impulsivity score < 17 points); and a high combined ADHD traits group (H-ADHD-C; inattention score ≥ 17 points and hyperactivity-impulsivity score ≥ 17 points) and its control group (L-C; inattention score < 17 points and hyperactivity-impulsivity score < 17 points). In this study, the Adult ADHD Self-Report Scale (ASRS) demonstrated good reliability and validity. The Cronbach’s alpha reliability coefficient was calculated to be 0.843, indicating high internal consistency. Additionally, the ASRS showed good criterion validity, with correlation coefficients of 0.440 (*N* = 277) and 0.549 (*N* = 277) when compared to the Wender Utah Rating Scale (WURS) and Barratt Impulsiveness Scale (BIS), respectively. These findings suggest that the ASRS is a reliable and valid measure for assessing adult ADHD symptoms.

### 2.5 Data analysis

Due to a significant difference in sample sizes between the high ADHD traits groups and control groups, it is likely that there is heterogeneity in variances between the two groups, which violates the assumption of homogeneity of variances required for independent samples *t*-test. Therefore, in the current study, when comparing differences between the two groups on ANT variables, the Bootstrap method from non-parametric tests is employed (Efron, 1979) (see Supplementary materials “2.5 Data Analysis”). To further investigate the reaction time-accuracy patterns in adults with high ADHD traits, we employed cluster analysis to explore the differences between the groups in accuracy and reaction time. Furthermore, from a continuous variable perspective, we initially conduct a correlation analysis to explore the relationship between ASRS total score and the three major indices. Subsequently, we establish a parallel mediation model “ADHD symptoms (total ASRS score)—specific attentional quality—overall attentional performance (BIS)” to further investigate the role of specific attentional quality in the relationship between ADHD symptoms and overall attentional performance (BIS).

### 2.6 Supplementary analysis

Considering that ADHD often co-occurs with anxiety depression and stress (Riglin et al., 2021; Gair et al., 2021; Frick et al., 2023), and anxiety and depression can affect attention performance or bias (Klein et al., 2018; Li and Li, 2022), we also collect participants’ anxiety and depression scores (see Supplementary materials “2.6 Supplementary Analysis”). In the supplementary analysis, we use SPSS26 and first conduct independent samples *t*-tests to explore whether there are significant differences in anxiety and depression scores between each ADHD trait group and the control group. If significant differences are found, we include anxiety and depression scores as covariates and use analysis of covariance (ANCOVA) to explore the differences in attention metrics among the ADHD trait groups.

## 3 Results

### 3.1 Attention network performance: adults with high ADHD traits primarily exhibit deficits in the alerting network

**Figure 1:**
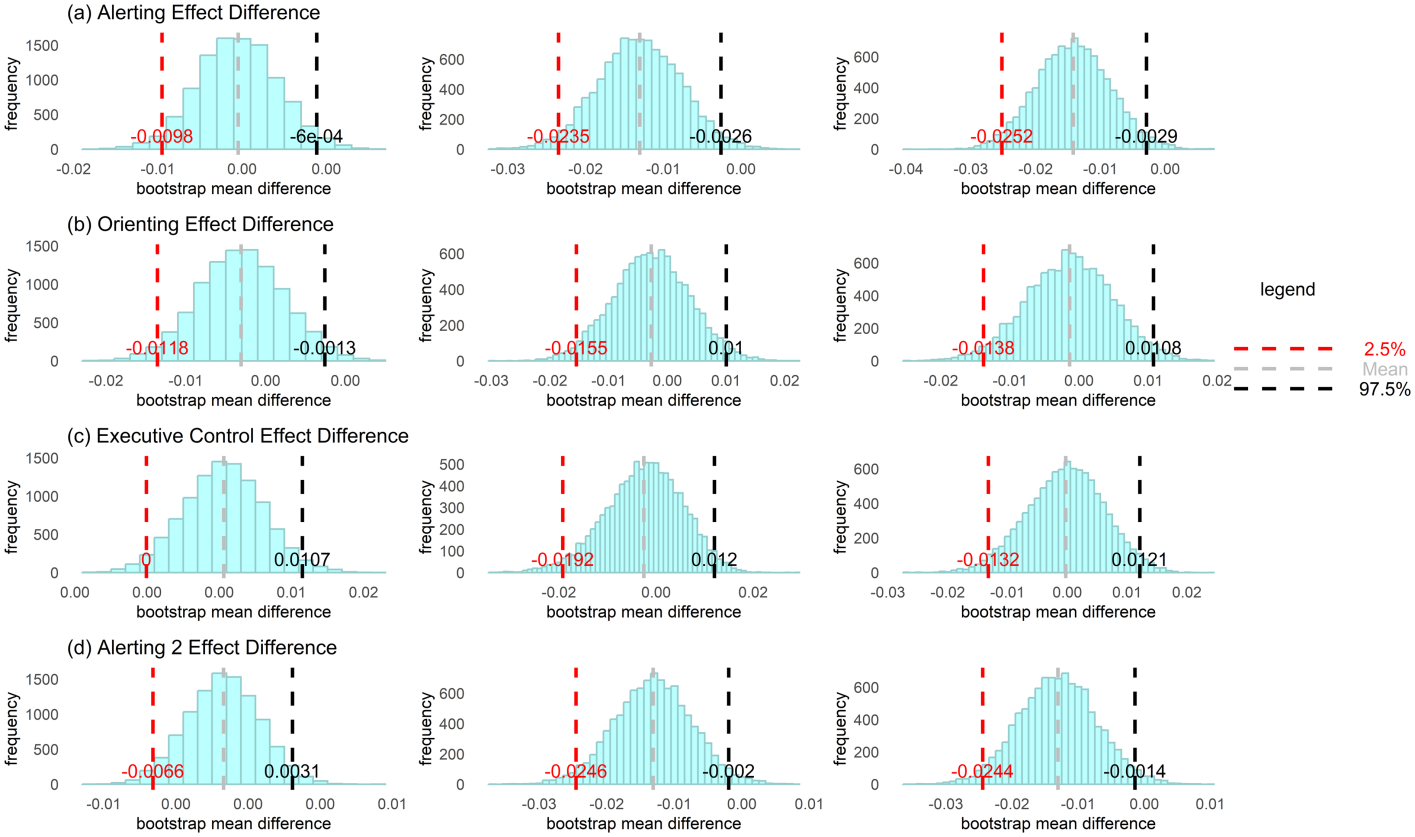
Group differences in attention networks between the ADHD and control groups (bootstrap resampled 10,000 times). Left column represents H-ADHD-I vs. control group, middle column represents H-ADHD-H vs. control group, and right column represents H-ADHD-C vs. control group.

Table 1 shows the means and standard deviations (SD) for the ANT variables in the high ADHD traits groups and the control groups. Further bootstrap tests (see Figure 1) indicate that H-ADHD-I group (95%CI, −0.010 to −0.001), H-ADHD-H group (95%CI, −0.024 to −0.003) and H-ADHD-C group (95%CI, −0.025 to −0.003) are all exhibit lower alerting effect compared to the control groups. The high inattention traits group (H-ADHD-I; 95%CI, −0.012 to −0.001) also demonstrates lower orienting effect than the control group. Only H-ADHD-H (95%CI, −0.025 to −0.002) and H-ADHD-C (95%CI, −0.024 to −0.001) groups show lower alerting 2 effect compared to the control groups, while the alerting 2 effect of H-ADHD-I (95%CI, −0.007 to 0.003) does not differ significantly from that of the control group. After controlling for the covariates of anxiety and depression, the differences between the H-ADHD-I group and the control group in alerting effect and orienting effect are no longer present (Supplementary Table S8). Overall, these results suggest that individuals with high-trait ADHD may exhibit deficits in sustained attention.

**Table 1:** Attention network performance in high ADHD traits groups compared to the control groups.

	Alerting effect (SD)	Orienting effect (SD)	Executive control effect (SD)	Alerting 2 effect (SD)
H-ADHD-I (*N* = 113)	−0.002 (0.040)	0.024 (0.049)	0.073 (0.073)	0.005 (0.041)
L-I (*N* = 226)	0.003 (0.036)	0.031 (0.040)	0.068 (0.041)	0.007 (0.037)
H-ADHD-H (*N* = 37)	−0.010 (0.053)	0.026 (0.047)	0.067 (0.065)	−0.006 (0.043)
L-H (*N* = 302)	0.003 (0.035)	0.029 (0.043)	0.070 (0.052)	0.008 (0.038)
H-ADHD-C (*N* = 34)	−0.011 (0.054)	0.030 (0.047)	0.068 (0.067)	−0.006 (0.044)
L-C (*N* = 223)	0.003 (0.036)	0.032 (0.040)	0.068 (0.041)	0.007 (0.038)

### 3.2 Attentional variability: adults with high ADHD traits exhibit high intra-individual coefficient of variation

Supplementary Table S2 shows the means and standard deviations (SD) for the ICV in the high ADHD traits groups and the control groups. To mitigate the potential influence of task impurity on the ICV metric, we compute ICV separately for congruent, incongruent, and neutral conditions in both the high ADHD traits and control groups, in addition to calculating the total ICV. Further bootstrap tests (see Supplementary Figure S3) indicate that all high ADHD traits groups exhibit higher ICV in all conditions compared to the control groups. Even after controlling for the covariates of anxiety and depression, this difference still exists (Supplementary Table S8). This indicates a deficiency in attentional stability among adults with high ADHD traits.

### 3.3 Overall attentional performance: adults with high attention deficit traits demonstrate high accuracy and characteristic responses with prolonged reaction times

Supplementary Table S3 shows the means and standard deviations (SD) for the BIS in the high ADHD traits groups and the control groups. Further bootstrap tests (see Supplementary Figure S4) indicate that high inattention traits group (95%CI, −0.402 to −0.108) and high combined ADHD traits group (95%CI, −0.787 to −0.091) exhibit lower BIS compared to the control groups. H-ADHD-H (95%CI, −0.646 to 0.035) shows no significant difference from the control group in terms of BIS. After controlling for the covariates of anxiety and depression, these differences persist.

To further analyze the response patterns in reaction time and accuracy between the two groups, we conduct a clustering analysis. Based on the data distribution and the results of Sum of Squared Errors within Clusters (SSE) (Supplementary Figure S5B) and silhouette coefficient values (Supplementary Table S4), we categorize the response patterns into three groups: High Accuracy-Low Reaction Time (High-Quick), Low Accuracy-Low Reaction Time (Low-Quick), and High Accuracy-High Reaction Time (High-Slow) (Supplementary Figure S5A). We then perform a chi-square test to compare the proportions of H-ADHD-I and H-ADHD-C in each response pattern category with the theoretical proportions. The results indicate that H-ADHD-I has a higher proportion than the theoretical proportion in the “High-Quick” group (*χ^2^* = 5.065, *p* = 0.024).

### 3.4 Mediation analysis: ADHD symptoms affect overall attentional performance through their impact on attentional variability

Supplementary Table S5 displays the Pearson’s correlation analysis results between the total ADHD score, three attention network effects, ICV, and BIS. Due to our aims and the significant correlations between total ADHD score and alerting effect, ICV (Log), and BIS, we established parallel mediation models “ADHD symptoms—alerting network—overall attentional performance” and “ADHD symptoms—attentional variability—overall attentional performance” (see Supplementary Table S6; Supplementary Figure S6). The alerting network does not mediate between ADHD symptoms and overall attentional performance, with an indirect effect of 0.009 and a 95% Bootstrap confidence interval of [−0.002, 0.026]. However, attentional variability does mediate between ADHD symptoms and overall attentional performance, with an indirect effect of −0.070 and a 95% Bootstrap confidence interval of [−0.134, −0.008]. The difference between the two indirect paths is 0.079, with a 95% Bootstrap confidence interval of [0.014, 0.145]. This indicates that, among specific attentional qualities, compared to the alerting network, attentional variability plays a more significant role in the impact of ADHD symptoms on overall attentional performance.

## 4 Discussion

This study investigates the attention characteristics of high ADHD traits individuals, and further explore how these specific attentional qualities influence overall attentional performance. Findings reveal distinct deficits in ANT for H-ADHD subgroups: H-ADHD-I shows deficits in attentional variability, and unique patterns in overall attentional performance. H-ADHD-H displays deficits in sustained attention and attentional variability, while H-ADHD-C exhibits deficits in sustained attention, attentional variability, and overall performance. Notably, attentional variability mediates the influence of ADHD symptoms on overall attentional performance, underscoring its crucial role. Overall, these results provide insights into the attentional characteristics of high ADHD traits adults, emphasizing the significance of attentional variability in shaping their overall performance.

All three groups, H-ADHD-I, H-ADHD-H, and H-ADHD-C, exhibit deficits in the alerting network before controlling for the covariates of anxiety and depression, indicating sustained attention difficulties. Further analysis shows that while H-ADHD-I performs similarly to the control group in alerting network 2, both H-ADHD-H and H-ADHD-C show poorer utilization of alerting cues. This suggests that H-ADHD-H and H-ADHD-C struggle with cue utilization, while the low alerting effect in H-ADHD-I is due to interference from distracting stimuli. Interestingly, when controlling for covariates, the differences between the H-ADHD-I group and the control group in alerting effect and orienting effect are no longer present. This suggests that the attention network in the H-ADHD-I group is more significantly influenced by anxiety and depression.

All three high ADHD traits groups exhibit higher ICV levels in various conditions compared to the control group (even after controlling the covariates of anxiety and depression), consistent with previous findings (Alemany et al., 2015; de Zeeuw et al., 2008). This indicates that high ADHD traits individuals have greater attentional variability, potentially related to deficits in sustained attention and stability. Our study highlights ICV’s significance as a sensitive and stable metric.

H-ADHD-I and H-ADHD-C show poorer overall attentional performance (BIS) compared to the control group. Furthermore, H-ADHD-I exhibits a pattern of “high accuracy-slow response,” suggesting the high accuracy may come at the cost of slower response times. However, from a practical standpoint, with willingness to invest time, H-ADHD-I can perform tasks as effectively as typical individuals, achieving a high level of accuracy. Additionally, both H-ADHD-H and H-ADHD-C have not exhibited abnormal patterns in terms of accuracy and response times, although H-ADHD-C exhibits a deficiency in BIS, possibly due to its mixed composition of H-ADHD-I and H-ADHD-H. Overall, these findings are crucial for motivating adults with high ADHD traits, and even adults with ADHD in general.

The parallel mediation analysis shows that the alerting network does not mediate between ADHD symptoms and overall attentional performance. This may be due to the dual effects of alerting, as it can lead to both faster response times and increased errors (Seli et al., 2012). On the other hand, attentional variability plays a significant mediating role, especially due to the substantial impact of ICV on BIS. As ADHD symptoms worsen, they are more likely to affect attentional stability, leading to poorer task performance (BIS). This emphasizes the importance of not only focusing on the attention network but also considering attentional variability in adult ADHD studies. It also highlights the need to address the impact of these variables on the social functioning of individuals with ADHD, ultimately guiding interventions to enhance their quality of life.

All three high ADHD traits groups surprisingly show no significant differences in the executive control network compared to the control group. This contrasts with previous findings in children with ADHD (Samyn et al., 2017; Yoon, 2010). However, deficits in the alerting network are consistently observed in both children and adults with high ADHD traits. This suggests that the development of other cognitive abilities in adults may compensate for executive control deficiencies during maturation. Nonetheless, foundational functions like sustained attention and alertness appear more resistant to natural compensation, indicating that the core deficit in ADHD symptoms lies in these foundational attentional skills, which are challenging to naturally compensate for in adulthood.

This study offers several notable contributions: (1) Investigating the mediating roles of attentional variability and the alerting network in ADHD symptoms’ impact on overall attentional performance holds theoretical and practical significance. The response pattern of individuals with H-ADHD-I, characterized as “high accuracy-slow response,” can be leveraged by educators to provide students with more time to complete tasks or tests. This strategy can help students improve their academic performance without compromising accuracy, thereby enhancing their confidence. (2) The use of BIS considers both speed and accuracy, addressing potential limitations of focusing solely on one metric. (3) The findings suggest that the alerting network, attentional variability (ICV), and BIS highlighted in the study can be used as supplementary diagnostic indicators. These measures can provide a more comprehensive assessment of ADHD characteristics, particularly in evaluating adults at risk for attention deficits, contributing to more precise diagnoses. In addition to assessment, career counselors can design suitable work paces and environments based on the attentional characteristics of ADHD individuals. For instance, they can arrange more flexible task schedules, allowing these individuals to leverage their high accuracy to compensate for slower response times. Cognitive training programs based on ICV and the alerting network can also be developed to help adults with ADHD enhance the persistence and stability of their attention.

## 5 Conclusion

This study systematically examines the attention network characteristics of adults with high ADHD traits and further, through parallel mediation models, elucidates the mediating role of specific attentional qualities in the impact of ADHD symptoms on overall attentional performance. The findings illuminate that H-ADHD individuals primarily display impairments in sustained attention (alerting network), attentional stability (ICV), and overall attentional performance. The overall attentional performance is exacerbated by the heightened attentional variability associated with ADHD symptoms. Given the influence of ADHD symptoms on daily functioning in adults, this study constructs a cognitive bridge between ADHD symptoms and general performance. It provides a unique contribution to our understanding of the cognitive mechanisms underlying the impact of ADHD symptoms on general performance. Additionally, it offers valuable insights and sensitive, stable indicators for the clinical diagnosis and intervention of adult ADHD, contributing to a deeper comprehension of the cognitive processes implicated in the manifestation and modulation of ADHD symptoms.

## Data Availability

The datasets presented in this study can be found in online repositories. The names of the repository/repositories and accession number(s) can be found at: https://osf.io/gk76e/.

## References

[ref1] Alemany S. Ribases M. Vilor-Tejedor N. Bustamante M. Sanchez-Mora C. Bosch R.. (2015). New suggestive genetic loci and biological pathways for attention function in adult attention-deficit/hyperactivity disorder. Am. J. Med. Genet. B Neuropsychiatr. Genet. 168, 459–470. doi: 10.1002/ajmg.b.32341 26174813

[ref2] American Psychiatric Association. (2013). Diagnostic and statistical manual of mental disorders (5th ed.). American Psychiatric Association Publishing.

[ref3] Anstey K. J. (1999). Sensorimotor variables and forced expiratory volume as correlates of speed, accuracy, and variability in reaction time performance in late adulthood. Aging Neuropsychol. Cogn. 6, 84–95. doi: 10.1076/anec.6.2.84.786

[ref4] Auerswald T. Hendker A. Ratz T. Lippke S. Pischke C. R. Peters M.. (2022). Impact of activity tracker usage in combination with a physical activity intervention on physical and cognitive parameters in healthy adults aged 60+: a randomized controlled trial. Int. J. Environ. Res. Public Health 19:3785. doi: 10.3390/ijerph19073785 35409466 PMC8997555

[ref5] Ayano G. Demelash S. Gizachew Y. Tsegay L. Alati R. (2023). The global prevalence of attention deficit hyperactivity disorder in children and adolescents: an umbrella review of meta-analyses. J. Affect. Disord. 339, 860–866. doi: 10.1016/j.jad.2023.07.071, PMID: 37495084

[ref6] Baggio S. Hasler R. Giacomini V. El-Masri H. Weibel S. Perroud N.. (2020). Does the continuous performance test predict ADHD symptoms severity and ADHD presentation in adults? J. Atten. Disord. 24, 840–848. doi: 10.1177/1087054718822060 30654686

[ref7] Bidwell L. C. Willcutt E. G. DeFries J. C. Pennington B. F. (2007). Testing for neuropsychological endophenotypes in siblings discordant for attention-deficit/hyperactivity disorder. Biol. Psychiatry 62, 991–998. doi: 10.1016/j.biopsych.2007.04.003, PMID: 17585884 PMC2687149

[ref8] Coll-Martin T. Carretero-Dios H. Lupianez J. (2021). Attentional networks, vigilance, and distraction as a function of attention-deficit/hyperactivity disorder symptoms in an adult community sample. Br. J. Psychol. 112, 1053–1079. doi: 10.1111/bjop.12513, PMID: 34089269

[ref9] de Zeeuw P. Aarnoudse-Moens C. Bijlhout J. Konig C. Uiterweer A. P. Papanikolau A.. (2008). Inhibitory performance, response speed, intraindividual variability, and response accuracy in ADHD. J. Am. Acad. Child Adolesc. Psychiatry 47, 808–816. doi: 10.1097/CHI.0b013e318172eee9, PMID: 18520957

[ref10] Dotare M. Bader M. Mesrobian S. K. Asai Y. Villa A. E. P. Lintas A. (2020). Attention networks in ADHD adults after working memory training with a dual n-Back task. Brain Sci. 10:715. doi: 10.3390/brainsci10100715, PMID: 33050115 PMC7600375

[ref6000] Efron B. (1979). 1977 rietz lecture – bootstrap methods – another look at the jackknife [Article]. Ann. Stat., 7, 1–26. doi: 10.1214/aos/1176344552

[ref11] Fan J. McCandliss B. D. Fossella J. Flombaum J. I. Posner M. I. (2005). The activation of attentional networks. NeuroImage 26, 471–479. doi: 10.1016/j.neuroimage.2005.02.004 15907304

[ref12] Fan J. McCandliss B. D. Sommer T. Raz A. Posner M. I. (2002). Testing the efficiency and independence of attentional networks. J. Cogn. Neurosci. 14, 340–347. doi: 10.1162/089892902317361886, PMID: 11970796

[ref13] Fan J. Posner M. (2004). Human attentional networks. Psychiatr. Prax. 31, S210–S214. doi: 10.1055/s-2004-828484, PMID: 15586312

[ref14] Faraone S. V. Asherson P. Banaschewski T. Biederman J. Buitelaar J. K. Ramos-Quiroga J. A.. (2015). Attention-deficit/hyperactivity disorder. Nat. Rev. Dis. Primers 1:1. doi: 10.1038/nrdp.2015.20 27189265

[ref15] Faraone S. V. Banaschewski T. Coghill D. Zheng Y. Biederman J. Bellgrove M. A.. (2021). The world federation of ADHD international consensus statement: 208 evidence-based conclusions about the disorder. Neurosci. Biobehav. Rev. 128, 789–818. doi: 10.1016/j.neubiorev.2021.01.022, PMID: 33549739 PMC8328933

[ref16] Frick M. A. Meyer J. Isaksson J. (2023). The role of comorbid symptoms in perceived stress and sleep problems in adolescent ADHD. Child Psychiatry Hum. Dev. 54, 1141–1151. doi: 10.1007/s10578-022-01320-z, PMID: 35094182 PMC10272236

[ref17] Gair S. L. Brown H. R. Kang S. Grabell A. S. Harvey E. A. (2021). Early development of comorbidity between symptoms of ADHD and anxiety. Res. Child Adolesc. Psychopathol. 49, 311–323. doi: 10.1007/s10802-020-00724-6, PMID: 33404952 PMC7878348

[ref18] Ghisletta P. Renaud O. Fagot D. Lecerf T. de Ribaupierre A. (2018). Age and sex differences in intra-individual variability in a simple reaction time task. Int. J. Behav. Dev. 42, 294–299. doi: 10.1177/0165025417739179

[ref19] Johnson B. P. Pinar A. Fornito A. Nandam L. S. Hester R. Bellgrove M. A. (2015). Left anterior cingulate activity predicts intra-individual reaction time variability in healthy adults. Neuropsychologia 72, 22–26. doi: 10.1016/j.neuropsychologia.2015.03.015 25791710

[ref20] Kim K. Kim H.-J. (2021). Normal executive attention but abnormal orienting attention in individuals with sluggish cognitive tempo. Int. J. Clin. Health Psychol. 21:100199. doi: 10.1016/j.ijchp.2020.08.003, PMID: 33363582 PMC7753035

[ref6001] Klein A. M. de Voogd L. Wiers R. W. Salemink E. (2018). Biases in attention and interpretation in adolescents with varying levels of anxiety and depression [Article]. Cogn. Emot., 32, 1478–1486. doi: 10.1080/02699931.2017.1304359 28366048

[ref21] Kooij J. J. S. Bijlenga D. Salerno L. Jaeschke R. Bitter I. Balazs J.. (2019). Updated European consensus statement on diagnosis and treatment of adult ADHD. Eur. Psychiatry 56, 14–34. doi: 10.1016/j.eurpsy.2018.11.001, PMID: 30453134

[ref22] Lampe K. Konrad K. Kroener S. Fast K. Kunert H. J. Herpertz S. C. (2007). Neuropsychological and behavioural disinhibition in adult ADHD compared to borderline personality disorder. Psychol. Med. 37, 1717–1729. doi: 10.1017/S0033291707000517, PMID: 17506923

[ref23] Levy F. Pipingas A. Harris E. V. Farrow M. Silberstein R. B. (2018). Continuous performance task in ADHD: is reaction time variability a key measure? Neuropsychiatr. Dis. Treat. 14, 781–786. doi: 10.2147/NDT.S158308, PMID: 29588592 PMC5858546

[ref24] Liesefeld H. R. Fu X. Zimmer H. D. (2015). Fast and careless or careful and slow? Apparent holistic processing in mental rotation is explained by speed-accuracy trade-offs. J. Exp. Psychol. Learn. Mem. Cogn. 41, 1140–1151. doi: 10.1037/xlm0000081, PMID: 25528084

[ref25] Liesefeld H. R. Janczyk M. (2019). Combining speed and accuracy to control for speed-accuracy trade-offs(?). Behav. Res. Methods 51, 40–60. doi: 10.3758/s13428-018-1076-x, PMID: 30022459

[ref26] Liesefeld H. R. Janczyk M. (2023). Same same but different: subtle but consequential differences between two measures to linearly integrate speed and accuracy (LISAS vs. BIS). Behav. Res. Methods 55, 1175–1192. doi: 10.3758/s13428-022-01843-2, PMID: 35595937 PMC10125931

[ref6002] Li S. Li X. (2022). The relationship between attentional bias, anxiety sensitivity, and depression and anxiety symptoms: evidence from the covid-19 pandemic in China [Article]. Front. Public Health, 10:832819. doi: 10.3389/fpubh.2022.832819 35211448 PMC8861176

[ref27] Loomans E. M. van der Stelt O. van Eijsden M. Gemke R. J. B. J. Vrijkotte T. G. M. Van den Bergh B. R. H. (2012). High levels of antenatal maternal anxiety are associated with altered cognitive control in five-year-old children. Dev. Psychobiol. 54, 441–450. doi: 10.1002/dev.20606 21953508

[ref28] Lundervold A. J. Adolfsdottir S. Halleland H. Halmoy A. Plessen K. Haavik J. (2011). Attention network test in adults with ADHD—the impact of affective fluctuations. Behav. Brain Funct. 7:27. doi: 10.1186/1744-9081-7-27, PMID: 21794128 PMC3168400

[ref29] Manly T. Davison B. Gaynord B. Greenfield E. Parr A. Ridgeway V.. (2004). An electronic knot in the handkerchief: "content free cueing" and the maintenance of attentive control. Neuropsychol. Rehabil. 14, 89–116. doi: 10.1080/09602010343000110

[ref30] McGee R. A. Clark S. E. Symons D. K. (2000). Does the Conners' continuous performance test aid in ADHD diagnosis? J. Abnorm. Child Psychol. 28, 415–424. doi: 10.1023/A:1005127504982 11100916

[ref31] Oberlin B. G. Alford J. L. Marrocco R. T. (2005). Normal attention orienting but abnormal stimulus alerting and conflict effect in combined subtype of ADHD. Behav. Brain Res. 165, 1–11. doi: 10.1016/j.bbr.2005.06.041, PMID: 16213034

[ref32] Pinto R. Asherson P. Ilott N. Cheung C. H. M. Kuntsi J. (2016). Testing for the mediating role of Endophenotypes using molecular genetic data in a twin study of ADHD traits. Am. J. Med. Genet. B Neuropsychiatr. Genet. 171, 982–992. doi: 10.1002/ajmg.b.32463, PMID: 27230021 PMC5031223

[ref33] Posner M. I. Petersen S. E. (1990). The attention system of the human brain. Annu. Rev. Neurosci. 13, 25–42. doi: 10.1146/annurev.ne.13.030190.000325 2183676

[ref34] Riglin L. Leppert B. Dardani C. Thapar A. K. Rice F. O'Donovan M. C.. (2021). ADHD and depression: investigating a causal explanation. Psychol. Med. 51, 1890–1897. doi: 10.1017/S0033291720000665, PMID: 32249726 PMC8381237

[ref35] Samyn V. Roeyers H. Bijttebier P. Wiersema J. R. (2017). Attentional networks in boys with ADHD or autism Spectrum disorder and the relationship with effortful control. J. Atten. Disord. 21, 228–239. doi: 10.1177/1087054712473183, PMID: 23382577

[ref36] Seli P. Cheyne J. A. Smilek D. (2012). Attention failures versus misplaced diligence: separating attention lapses from speed-accuracy trade-offs. Conscious. Cogn. 21, 277–291. doi: 10.1016/j.concog.2011.09.017 22001770

[ref37] Song P. Zha M. Yang Q. Zhang Y. Li X. Rudan I.. (2021). The prevalence of adult attention-deficit hyperactivity disorder: a global systematic review and meta-analysis. J. Glob. Health 11:11. doi: 10.7189/jogh.11.04009 PMC7916320 33692893

[ref38] Vandierendonck A. (2017). A comparison of methods to combine speed and accuracy measures of performance: a rejoinder on the binning procedure. Behav. Res. Methods 49, 653–673. doi: 10.3758/s13428-016-0721-5, PMID: 26944576

[ref39] Vazquez-Marrufo M. Garcia-Valdecasas Colell M. Galvao-Carmona A. Sarrias-Arrabal E. Tirapu-Ustarroz J. (2019). The attention network test in the study of cognitive impairment of ADHD patients. Rev. Neurol. 69, 423–432. doi: 10.33588/rn.6910.2019202, PMID: 31713229

[ref40] Wallace S. Morton S. E. Linscott R. J. (2019). Relationships between intra-individual variability and subclinical psychosis. Psychiatry Res. 281:112592. doi: 10.1016/j.psychres.2019.112592, PMID: 31586835

[ref9002] Yeh C-B Gau SS-F Kessler RC Wu Y-Y. (2008). Psychometric properties of the Chinese version of the adult ADHD Self-report Scale [Article]. Int J Methods Psychiatr. Res, 17, 45–54. doi: 10.1002/mpr.241 18286465 PMC6878254

[ref41] Yoon B. S. (2010). The possibility of meditation based intervention on ADHD. J. Emot. Behav. Disord. 26, 121–143.

[ref42] Zamani Sani S. H. Fathirezaie Z. Sadeghi-Bazargani H. Badicu G. Ebrahimi S. Grosz R. W.. (2020). Driving accidents, driving violations, symptoms of attention-deficit-hyperactivity (ADHD) and attentional network tasks. Int. J. Environ. Res. Public Health 17:5238. doi: 10.3390/ijerph17145238, PMID: 32698490 PMC7400088

