# Supplementary material for: The attention network characteristics of adults with high ADHD traits: low stability, boost accuracy by sacrificing response time
Source: Front Psychol. 2024 Dec 18;15:1477581. doi: 10.3389/fpsyg.2024.1477581 (PMC11688296; doi:10.3389/fpsyg.2024.1477581)
Supplement: Supplementary file 1 [file Table_1.DOCX]

Supplementary Material

**2.6 Supplementary analysis**

**Measuring anxiety, depression and perceived stress:** We use the Trait Anxiety Inventory (TAI) subscale from the State-Trait Anxiety Inventory (STAI) to measure participants' anxiety levels. This subscale consists of 20 items, rated on a 1-4 scale. Higher scores on this subscale indicate higher levels of anxiety in the participants (1, 2). The Self-Rating Depression Scale (SDS) is used to measure participants' depression scores. This scale is rated on a 1-4 scale, with higher total scores indicating more severe depressive symptoms (3, 4). The perceived stress scale (PSS) is used to measure participants' perceived stress. This scale is rated on a 0-4 scale, with higher total scores indicating more perceived stress (5).

**3. results**

**Supplementary results**

As shown in Table S6, the results of the independent samples t-test indicate that H-ADHD-I, H-ADHD-H, and H-ADHD-C each exhibit significant differences from their respective control groups in terms of anxiety and depression scores. Therefore, the significant different scores (anxiety and depression scores) are included as covariates in subsequent analysis of covariance (ANCOVA). The results of ANCOVA are presented in Table S7, and the findings reveal that except for the absence of differences in alerting effect and orienting effect for H-ADHD-I, other results are consistent with those obtained before controlling for covariates.

## Supplementary Figures


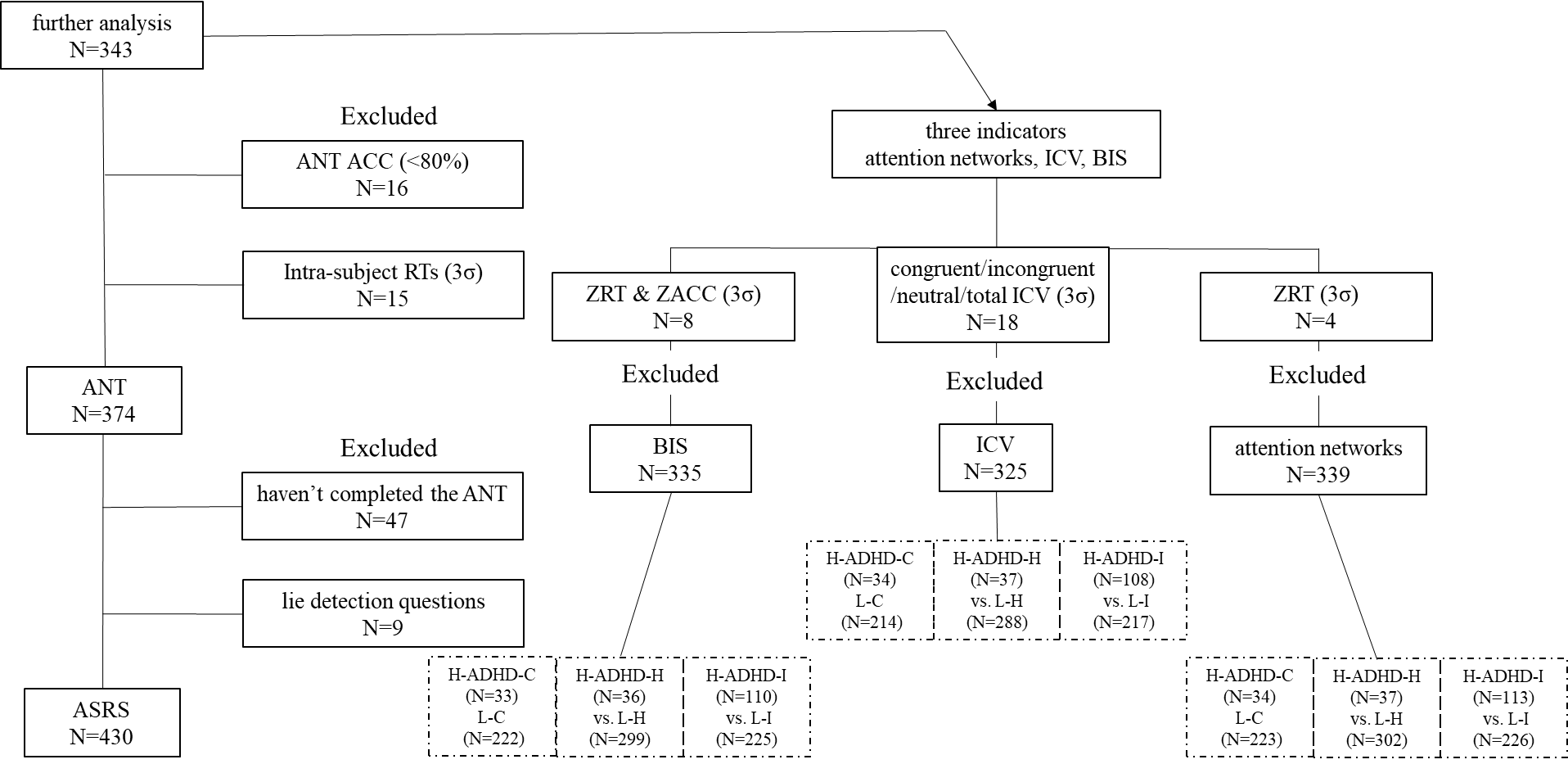


**Figure S1.** Participant Screening Flowchart


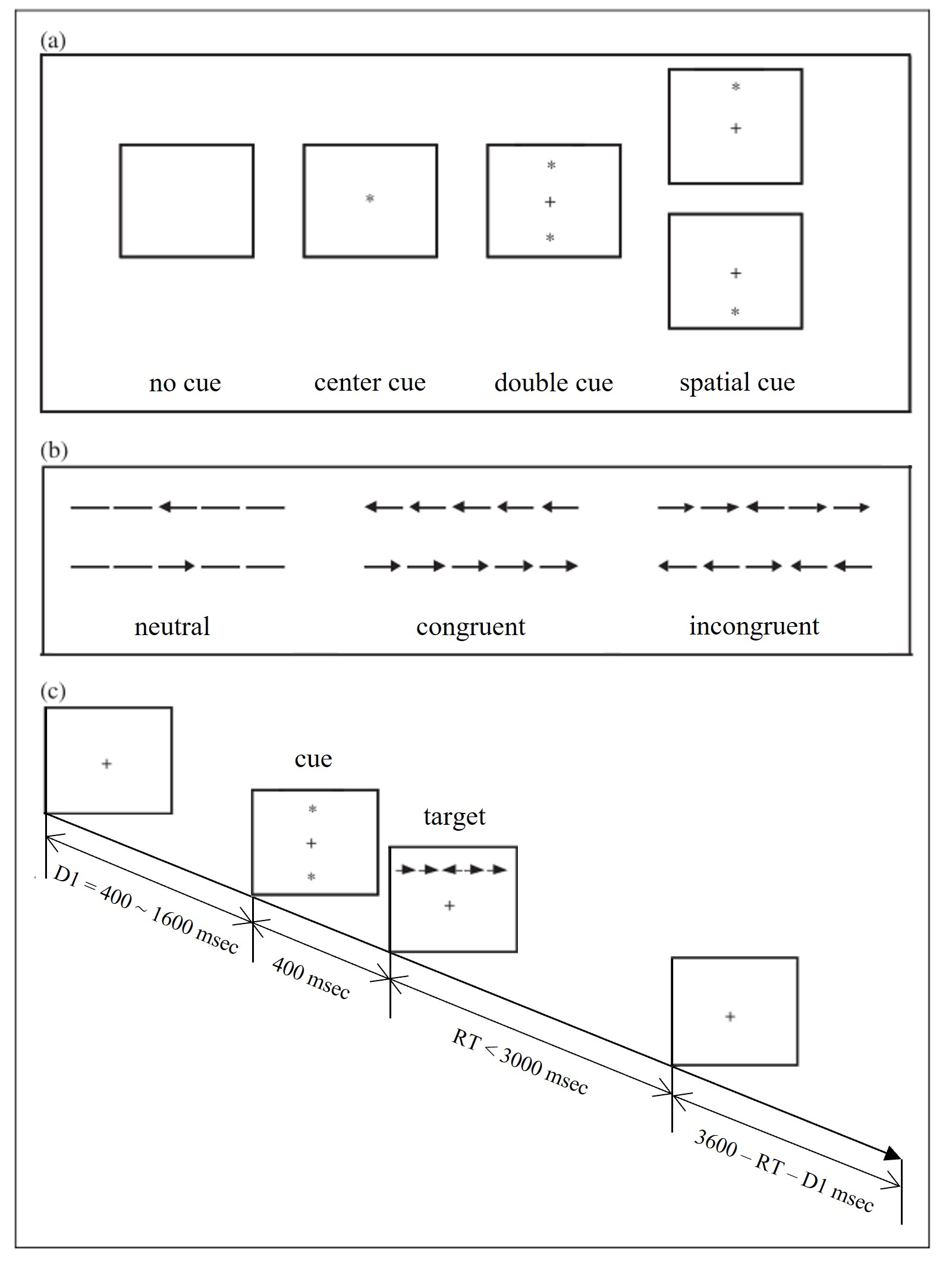


**Figure S2.** Experimental procedure. (a) The four cue conditions; (b) The six stimuli used in the present experiment; and (c) An example of the procedure.


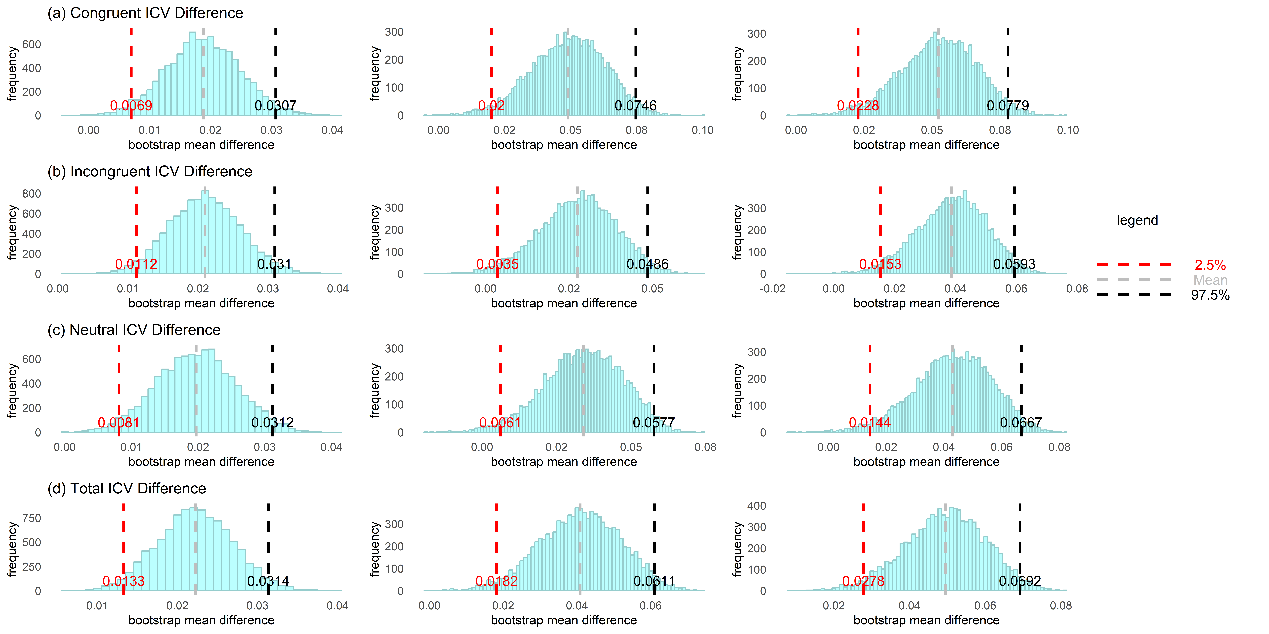


**Figure S3.** Group differences in ICVs between the ADHD and control groups (Bootstrap resampled 10,000 times)

Note: Left column represents H-ADHD-I vs. control group, middle column represents H-ADHD-H vs. control group, and right column represents H-ADHD-C vs. control group.


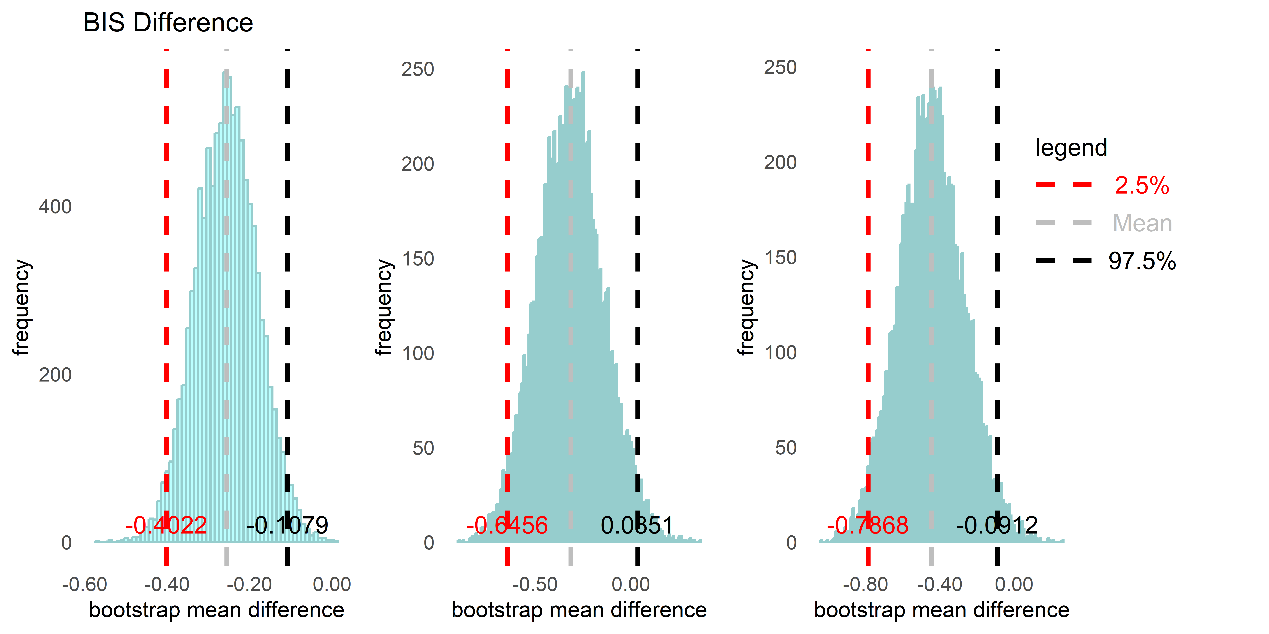


**Figure S4.** Group differences in BIS between the ADHD and control groups (Bootstrap resampled 10,000 times)

Note: Left column represents H-ADHD-I vs. control group, middle column represents H-ADHD-H vs. control group, and right column represents H-ADHD-C vs. control group.


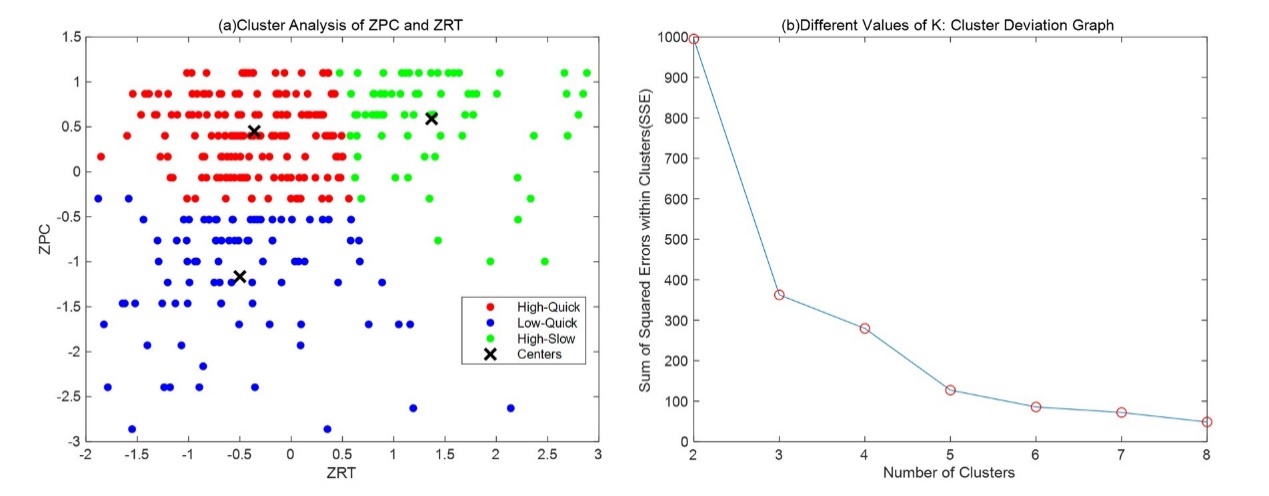


**Figure S5.** Cluster analysis and Sum of Squared Errors within Clusters (SSE) results


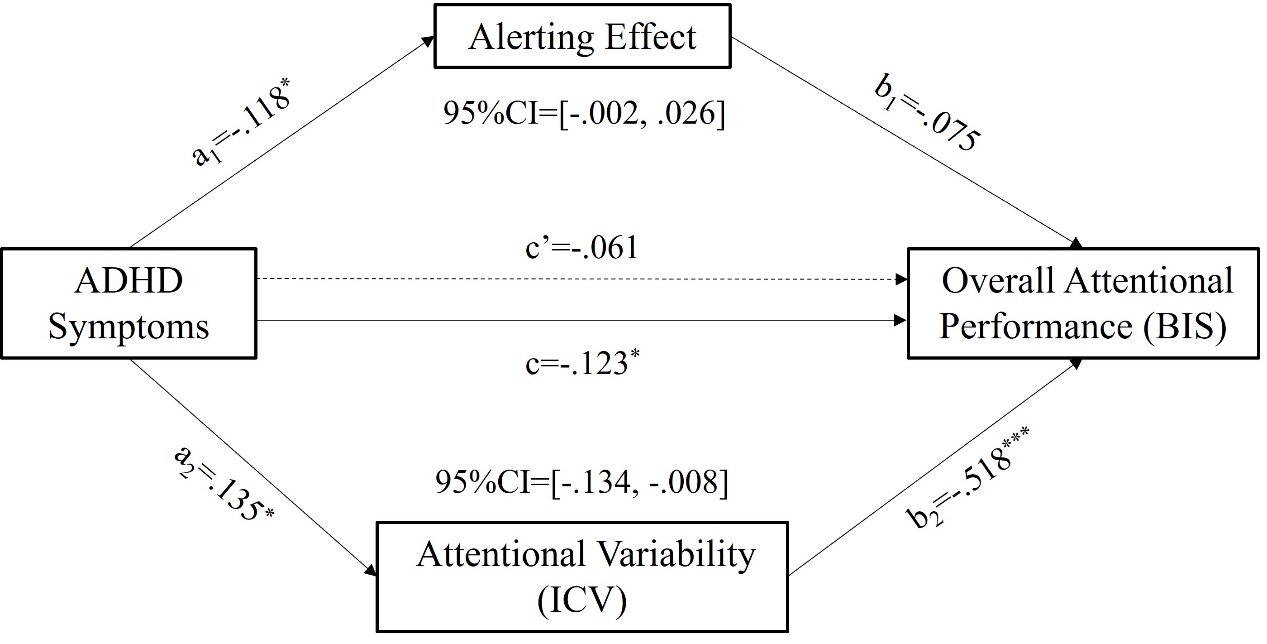


**Figure S6.** Parallel mediation model

**Table S1.** Demographic table

| Average annual household income (*yuan*) | N |
| --- | --- |
| <5000 | 69 |
| 5001~25000 | 114 |
| 25001~45000 | 60 |
| 45001~65000 | 43 |
| 65001~85000 | 18 |
| 85001~105000 | 12 |
| >105000 | 23 |
| NA | 4 |

Note: NA represents missing value.

**Table S2.** Intra-individual Coefficient of Variation in High ADHD traits Groups Compared to the Control Groups

|  | Congruent ICV  (SD) | Incongruent ICV  (SD) | Neutral ICV  (SD) | Total ICV  (SD) |
| --- | --- | --- | --- | --- |
| H-ADHD-I  (N=108) | .249(.114) | .210(.088) | .200(.107) | .240(.093) |
| L-I  (N=217) | .230(.088) | .189(.074) | .181(.086) | .218(.199) |
| H-ADHD-H (N=37) | .280(.133) | .221(.101) | .217(.136) | .261(.106) |
| L-H  (N=288) | .231(.091) | .193(.076) | .183(.087) | .220(.072) |
| H-ADHD-C (N=34) | .282(.139) | .229(.102) | .224(.139) | .267(.109) |
| L-C  (N=214) | .230(.089) | .190(.074) | .181(.087) | .218(.069) |

**Table S3.** Balanced Integration Score in High ADHD traits Groups Compared to the Control Groups

|  | Balanced Integration Score (SD) |
| --- | --- |
| H-ADHD-I (N=110) | -.087(1.188) |
| L-I (N=225) | .169(1.088) |
| H-ADHD-H (N=36) | -.198(1.153) |
| L-H (N=299) | .119(1.120) |
| H-ADHD-C (N=33) | -.283(1.122) |
| L-C (N=222) | .161(1.086) |

**Table S4.** correlation analysis (Standardized)

|  | M(SD) | 1 | 2 | 3 | 4 | 5 | 6 |
| --- | --- | --- | --- | --- | --- | --- | --- |
| 1. total ADHD score | 26.06(7.92) | - |  |  |  |  |  |
| 2. alerting effect | .003(.036) | -.118^*^ | - |  |  |  |  |
| 3. orienting effect | .029(.039) | -.081 | .132^*^ | - |  |  |  |
| 4. executive control | .067(.043) | -.043 | -.054 | .086 | - |  |  |
| 5. ICV(Log) | .223(.076) | .135^*^ | .048 | -.038 | .144^*^ | - |  |
| 6. BIS | .181(1.026) | -.123^*^ | -.092 | -.001 | -.097 | -.530^**^ | - |

Note: ^*^*p* < .05. ^**^*p* < .01.

**Table S5.** mediation analysis

| *dependent variable* | *predictor variable* | *β* | *t* | *R* | *R^2^* | *F* |
| --- | --- | --- | --- | --- | --- | --- |
| ZBIS | ZTotal | -.061 | -1.265 | .537 | .289 | 42.315 |
|  | ZAlerting | -.075 | -1.552 |  |  |  |
|  | ZLogICV | -.518 | -10.735^***^ |  |  |  |
| ZLogICV | ZTotal | .135 | 2.423^*^ | .135 | .018 | 5.871 |
| ZAlerting | ZTotal | -.118 | -2.114^*^ | .118 | .014 | 4.470 |

Note: Z represents the standardized value. ^*^*p* < .05. ^**^*p* < .01.

**Table S6.** results of the independent samples t-test

| Scale | Group | *M(SD)* | *t* | *p* | *95%CI* |
| --- | --- | --- | --- | --- | --- |
| TAI | H-ADHD-I(N=112) | 46.411(7.409) | 5.640 | .000 | (3.098,6.417) |
|  | L-I (N=225) | 41.653(7.237) |  |  |  |
|  | H-ADHD-H(N=37) | 47.081(6.841) | 3.301 | .001 | (1.746,6.896) |
|  | L-H (N=300) | 42.760(7.589) |  |  |  |
|  | H-ADHD-C(N=34) | 46.588(6.706) | 3.896 | .000 | (2.514,7.653) |
|  | L-C (N=222) | 41.505(7.140) |  |  |  |
| SDS | H-ADHD-I(N=112) | 47.741(8.801) | 5.556 | .000 | (3.249,6.811) |
|  | L-I (N=225) | 42.711(7.299) |  |  |  |
|  | H-ADHD-H(N=37) | 48.081(7.131) | 2.952 | .003 | (1.386,6.923) |
|  | L-H (N=300) | 43.927(8.183) |  |  |  |
|  | H-ADHD-C(N=34) | 47.824(6.917) | 3.953 | .000 | (2.622,7.827) |
|  | L-C (N=222) | 42.599(7.213) |  |  |  |
| PSS | H-ADHD-I(N=111) | 28.351(6.243) | 1.648 | .100 | (-.227,2.567) |
|  | L-I (N=221) | 27.181(6.034) |  |  |  |
|  | H-ADHD-H(N=36) | 28.556(6.553) | .961 | .342 | (-1.212,3.418) |
|  | L-H (N=296) | 27.453(6.066) |  |  |  |
|  | H-ADHD-C(N=33) | 28.515(6.764) | 1.090 | .282 | (-1.161,3.879) |
|  | L-C (N=218) | 27.156(6.057) |  |  |  |

Note: TAI: anxiety score; SDS: depression score; PSS: perceived stress score.

**Table S7.** results of the analysis of covariance (ANCOVA)

| Scale | Group | *M(SD)* | *F* | *p* | *η^2^* |
| --- | --- | --- | --- | --- | --- |
| alerting effect | H-ADHD-I(N=112) | -.002(.041) | 1.745 | .187 | .005 |
|  | L-I (N=225) | .003(.036) |  |  |  |
|  | H-ADHD-H(N=37) | -.010(.053) | 4.214 | .041 | .012 |
|  | L-H (N=300) | .003(.035) |  |  |  |
|  | H-ADHD-C(N=34) | -.011(.054) | 4.648 | .032 | .018 |
|  | L-C (N=222) | .003(.036) |  |  |  |
| orienting effect | H-ADHD-I(N=112) | .025(.048) | 2.133 | .145 | .006 |
|  | L-I (N=225) | .031(.040) |  |  |  |
|  | H-ADHD-H(N=37) | .026(.047) | .230 | .632 | .001 |
|  | L-H (N=300) | .029(.043) |  |  |  |
|  | H-ADHD-C(N=34) | .030(.047) | .024 | .876 | .000 |
|  | L-C (N=222) | .032(.040) |  |  |  |
| executive control  effect | H-ADHD-I(N=112) | .073(.073) | .447 | .504 | .001 |
|  | L-I (N=225) | .068(.041) |  |  |  |
|  | H-ADHD-H(N=37) | .067(.065) | .201 | .655 | .001 |
|  | L-H (N=300) | .070(.052) |  |  |  |
|  | H-ADHD-C(N=34) | .068(.068) | .050 | .822 | .000 |
|  | L-C (N=222) | .068(.041) |  |  |  |
| Total ICV | H-ADHD-I(N=107) | .240(.093) | 5.224 | .023 | .016 |
|  | L-I (N=216) | .218(.068) |  |  |  |
|  | H-ADHD-H(N=37) | .261(.106) | 8.240 | .004 | .025 |
|  | L-H (N=286) | .221(.073) |  |  |  |
|  | H-ADHD-C(N=34) | .268(.109) | 11.843 | .001 | .046 |
|  | L-C (N=213) | .218(.069) |  |  |  |
| BIS | H-ADHD-I(N=109) | -.102(1.184) | 3.485 | .063 | .010 |
|  | L-I (N=224) | .169(1.090) |  |  |  |
|  | H-ADHD-H(N=36) | -.198(1.153) | 2.012 | .157 | .006 |
|  | L-H (N=297) | .115(1.121) |  |  |  |
|  | H-ADHD-C(N=33) | -.283(1.122) | 4.497 | .035 | .018 |
|  | L-C (N=221) | .162(1.088) |  |  |  |

1. Shek DTL. RELIABILITY AND FACTORIAL STRUCTURE OF THE CHINESE VERSION OF THE STATE-TRAIT ANXIETY INVENTORY. Journal of Psychopathology and Behavioral Assessment. 1988;10(4):303-17.

2. Spielberger CD, Gorsuch RL, Lushene RE, editors. Manual for the State-Trait Anxiety Inventory1970.

3. Zung WWK. A SELF-RATING DEPRESSION SCALE. Archives of general psychiatry. 1965;12(1):63-70.

4. Zung WWK, Richards CB, Short MJ. SELF-RATING DEPRESSION SCALE IN AN OUTPATIENT CLINIC - FURTHER VALIDATION OF SDS. Archives of general psychiatry. 1965;13(6):508-&.

5. Cohen S, Kamarck T, Mermelstein R. A GLOBAL MEASURE OF PERCEIVED STRESS. Journal of Health and Social Behavior. 1983;24(4):385-96.
